# Human Hemangioblast-Derived Mesenchymal Stem Cells Promote Islet Engraftment in a Minimal Islet Mass Transplantation Model in Mice

**DOI:** 10.3389/fmed.2021.660877

**Published:** 2021-04-15

**Authors:** Suzanne Bertera, Michael F. Knoll, Carmela Knoll, Hidetaka Hara, Erin A. Kimbrel, Nickolas A. Kouris, Robert Lanza, Brett E. Philips, Yesica Garciafigueroa, Nick Giannoukakis, David K. C. Cooper, Massimo Trucco, Rita Bottino

**Affiliations:** ^1^Institute of Cellular Therapeutics, Allegheny-Singer Research Institute, Allegheny Health Network, Pittsburgh, PA, United States; ^2^Department of Surgery, Xenotransplantation Program, University of Alabama at Birmingham, Birmingham, AL, United States; ^3^Astellas Institute for Regenerative Medicine, Westborough, MA, United States; ^4^Department of Biological Sciences, Carnegie Mellon University, Pittsburgh, PA, United States

**Keywords:** hemangioblast-derived mesenchymal cell, human embryonic stem cell, mesenchymal stem cell, type 1 diabetes, islet transplantation, minimal islet mass model

## Abstract

Islet transplantation can restore glycemic control in patients with type 1 diabetes. Using this procedure, the early stages of engraftment are often crucial to long-term islet function, and outcomes are not always successful. Numerous studies have shown that mesenchymal stem cells (MSCs) facilitate islet graft function. However, experimental data can be inconsistent due to variables associated with MSC generation (including donor characteristics and tissue source), thus, demonstrating the need for a well-characterized and uniform cell product before translation to the clinic. Unlike bone marrow- or adipose tissue-derived MSCs, human embryonic stem cell-derived-MSCs (hESC-MSCs) offer an unlimited source of stable and highly-characterized cells that are easily scalable. Here, we studied the effects of human hemangioblast-derived mesenchymal cells (HMCs), (i.e., MSCs differentiated from hESCs using a hemangioblast intermediate), on islet cell transplantation using a minimal islet mass model. The co-transplantation of the HMCs allowed a mass of islets that was insufficient to correct diabetes on its own to restore glycemic control in all recipients. Our *in vitro* studies help to elucidate the mechanisms including reduction of cytokine stress by which the HMCs support islet graft protection *in vivo*. Derivation, stability, and scalability of the HMC source may offer unique advantages for clinical applications, including fewer islets needed for successful islet transplantation.

## Introduction

Islet transplantation by portal vein infusion restores glycemic balance and reduces hypoglycemic episodes in patients with type 1 diabetes that have difficulty controlling their symptoms using exogenous insulin (1–3). However, two or more pancreas donors are often necessary to achieve therapeutic outcomes (1, 2, 4). A significant loss of islets occurs soon after transplantation resulting from several factors including complement activation and coagulation, characterized as the instant blood-mediated inflammatory reaction (IBMIR), which becomes stronger as the transplantation scenario moves from autologous to xenogeneic (5–9). Transplantation of islets under the kidney capsule may reduce severity of IBMIR, although loss due to inflammation, lack of nutrients, and hypoxia may still result in inconsistent engraftment (10, 11). Our own *in vitro* data suggest that an immune response also plays a role in the early loss of islets (7, 9).

Long-term graft survival is likely if the graft is able to overcome peri-transplant insults and conserve sufficient islet mass (12). Therefore, investigators have sought to develop strategies to reduce early islet loss, including the co-transplantation of islets with mesenchymal stem cells (MSCs), that have been shown to provide protection for the graft in the crucial early stages after transplantation (13, 14).

MSCs are fibroblast-like multipotent cells defined as (i) plastic-adherent when maintained in standard culture conditions; (ii) expressing surface markers CD105, CD73 and CD90; (iii) lacking the expression of CD45, CD34, CD14 or CD11b, CD79α or CD19 and HLA-DR; and (iv) possessing the ability to differentiate to osteoblasts, adipocytes and chondroblasts *in vitro* (15). They have the ability to localize to inflamed tissue and facilitate tissue repair by secreting an assortment of cytokines, trophic factors, and anti-inflammatory molecules in response to micro-environmental stimuli (16); and have been extensively evaluated in clinical trials to treat a wide range of medical conditions (17), including diabetes (18). Typically, MSCs have been derived from human bone marrow, adipose or other tissues where donor characteristics, including age, gender, and health status, can affect cell potency and play critical roles in how effective MSCs are at enhancing islet engraftment (19, 20). The necessity to culture and expand these cell lines in order to produce a sufficient number of MSCs may also affect product potency and impact therapeutic effectiveness of individual batches (21). A meta-analysis of clinical studies shows that MSCs can be safely used in humans (22), yet efficacy data involving MSCs is often lacking, inconclusive, contradictory, or irreproducible, which limits their potential clinical impact (23).

Unlike marrow- or adipose-derived MSCs, HMCs can be expanded in unlimited quantity with consistent quality. HMCs exhibit characteristics similar to those of human bone marrow-derived MSCs, including immunomodulatory properties, with the advantage of higher proliferative capacity, making them a viable cell source for clinical application (21, 24, 25). The HMCs used in our study (21, 26, 27) were derived from a hESC line used in previous clinical trials where they were differentiated into retinal pigment epithelium to treat macular degeneration (28, 29). These HMCs display immunomodulatory properties and therapeutic activity in preclinical models for diseases such as lupus, uveitis, multiple sclerosis, and fistulizing Crohn's Disease (21, 30–33). We have used these cells for the first time to study their effect in a preclinical model of diabetes.

The goal of our study was to assess the effect of these HMCs on the outcome of islet transplantation in a mouse model. Our working hypothesis was that co-transplanted HMCs allow a minimal mass of islets to restore normoglycemia when islets alone cannot.

## Materials and Methods

### Experimental Design

We transplanted mouse islets under the kidney capsule of immunodeficient mice rendered diabetic with streptozotocin, either (i) alone, (ii) with HMCs, or (iii) with human dermal fibroblasts (HDFs), a similar cell type to MSCs not expected to protect the islet graft.

A minimal islet mass model was determined by transplanting incrementally fewer islets in diabetic mouse recipients, and by subsequently assessing metabolic control of the recipients. In our experiment, 100 isolated islets from a BALB/c mouse line proved to be an insufficient islet mass to correct glycemia and, therefore, this number was set as the baseline marginal mass to test whether the addition of HMCs would enhance islet graft performance.

The decision to use immunodeficient mice as recipients was made in order to dispel any potentially confounding immunological issues concerning immunosuppressive therapy in cross-species transplantation, as well as to establish and standardize a model that can be applied to islet xenotransplantation studies. The ratio of 500 HMCs/islet used in the study was based on published work (34).

### Animals

The mice in this study were approved for use by the Allegheny Health Network Institutional Animal Care and Use Committee (IACUC) and were cared for in accordance with the Guide for the Care and Use of Laboratory Animals and NIH guidelines. Mouse islet recipients: J:Nu nude homozygous males, 5–6 weeks old; mouse islet donors: BALB/cJ males, 8–10 weeks old (Jackson Laboratory, Bar Harbor, ME).

### Generation of HMCs

The starting hESC line was MA09, a US FDA-approved hESC line established using single blastomere technology (26). To generate HMCs, MA09 hESCs were first differentiated into embryoid bodies, then into hemangioblasts, and subsequently into HMCs, as previously described (21) and then cryopreserved.

### HMCs Culture

Frozen HMCs (from passage 4) and HDFs were shipped from the Astellas Institute for Regenerative Medicine (Marlborough, MA) to the Institute of Cellular Therapeutics (Pittsburgh, PA) in a cryogenic dry shipper.

Prior to use, they were thawed and cultured in MSC-maintenance medium (αMEM, 20% FBS, 1X pen/strep, 1X non-essential-AA, 1X l-glutaMAX) for 4 days in a humidified CO_2_ incubator at 37°C. These conditions ensure that the cells maintain the phenotype and functional characteristics previously assessed and determined (21). The cells were harvested with TrypLE (Thermo Fisher Scientific, Waltham, MA), washed with MSC medium, counted, and kept on ice until the time of transplant.

To further determine the specificity of the effect of HMCs on islet transplantation, HDFs were (i) co-transplanted with islets in parallel with (ii) islets transplanted alone, and (iii) islets plus HMCs. Cell viability was assessed with Trypan Blue dye exclusion test (Life-Technologies Gibco, Carlsbad, CA) after thawing and on the day of use, and was estimated to be >90% in all experiments.

### Islet Isolation

Mouse islets were isolated using a collagenase-based digestion (Collagenase type V, Sigma-Aldrich, St. Louis, MO), as previously published (35). Briefly, immediately after euthanasia, 2–3 ml cold collagenase solution (1.95 mg/ml in HBSS) was injected into the pancreas through the common bile duct. The fully inflated pancreas was excised and incubated for 20 min at 37°C in a tissue culture flask, then shaken for 5 s to break up the tissue. The digested tissue was washed 4X with cold HBSS supplemented with 0.2% BSA (HBSS/BSA), and the islets purified on a Ficoll (Type 400) gradient (Amersham, Little Chalfont, UK). Following gradient separation and two washes in HBSS/BSA, the islets were hand-picked and plated in culture dishes in CMRL 1066 medium (Mediatech, Manassas, VA) supplemented with 10% heat-inactivated fetal bovine serum (Invitrogen, Carlsbad, CA), 100 units/ml penicillin, 0.1 mg/ml streptomycin, and 2 mmol/l L-glutamine (Life Technologies, Grand Island, NY). All islets were used between 24 and 48 h after isolation. Each islet batch was composed of a pool of 8–10 mouse donors. Islet viability was determined to be >90% in each batch.

### *In vitro* Viability and Insulin Secretion Experiments

To determine whether HMCs affect islet insulin secretion and viability, we established co-cultures of islets and HMCs or HDFs (in numeric combination that reflect the *in vivo* experiments, thus 25 BALB/c mouse islets with or without 12,500 HMCs or HDFs) in two different settings—(i) direct contact between islets and cells, and (ii) indirect contact using Transwells (Sigma-Aldrich) with islets in the top chamber and HMCs or HDFs in the bottom. A mix of media (50% medium formulation for islets and 50% medium formulation for HMC cells, as described in Methods) was used. This medium combination does not negatively affect islet or HMC/HDF viability.

Triplicates of 25 islets/condition (islets alone, or plus HMCs, or plus HDFs) were studied in two independent experiments. Each islet batch (one for each of the two independent experiments) was obtained from a pool of 10 donor mice. HMC and HDF cells were obtained from multiple vials. After 48 h of co-culture, viability was assessed using a Calcein AM and Propidium iodide method (35). Glucose-stimulation of insulin secretion (GSIS) was carried out using a static incubation method (36, 37). Insulin concentration of the resulting medium was determined by ELISA (ALPCO, Salem, NH).

A second *in vitro* experiment was performed to determine if HMCs protect islets from the effect of inflammatory cytokines. Triplicates of 25 BALB/c islets with and without HMCs or HDFs (direct contact) were cultured for 24 h in the presence of a cocktail of IL-1 beta, TNF-alpha and INF-gamma at the concentration of 50 U/ml, 10^3^U/ml, and 10^3^U/ml respectively (36). Following culture, islet viability was determined and supernatant from the cultures was collected for measurement of insulin concentration. Islets were then subjected to GSIS as previously described.

### Induction of Diabetes in Transplant Recipients

Streptozotocin (Sigma-Aldrich) was freshly prepared with sterile saline at a concentration of 50 mg/ml and kept at 4°C until injection. Recipients were administered 240 mg/kg at least 5 days before islet transplantation (35). Mice becoming hyperglycemic (350–500 mg/dl on at least two consecutive blood glucose readings) were included in the study, and maintained on insulin therapy (0.5–1.0U daily, Humulin N, Lilly USA, LLC, Indianapolis, IN) for glycemic control, and given fluids as necessary to prevent dehydration.

### Islet Transplantation

Prior to transplantation, islets were handpicked, and only those with a diameter between 100 and 200 μm were used. Islet aliquots were counted by independent operators and randomly assigned to the experimental and control groups. When HMCs or HDFs were to be co-transplanted, the cells (in saline solution) were mixed with the islets just prior to transplantation. Mice were stratified based on their blood glucose levels on pre-transplant day-1 (350–400 mg/dl and >400 mg/dl) then randomly placed into transplant groups (islets alone or with either HMCs or HDFs) so that a representative mix of blood glucose levels were in each group.

For surgery, recipient mice were anesthetized with ketamine (100 mg/kg; Ketalar, Par Pharmaceutical, Spring Valley, NY) and xylazine (8 mg/kg; Pharm Eco Laboratories, Devens MA). After shaving and disinfecting the surgical site with betadine, a small incision (1 cm) was made in the flank of the mouse about one centimeter to the left of the midline at the level of the kidney. Another incision was made through the abdominal wall, the kidney externalized and a small opening made in the capsule at the distal pole of the kidney. The islets (alone or mixed with HMCs or HDFs) were gathered into an 8–10cm length of tubing (PE50, Harvard Apparatus, Holliston, MA) in an initial volume of 3–4 μl, and inserted through the capsule opening into the space between capsule and kidney. The islets/cells were slowly expelled as the tubing was withdrawn. The kidney was then replaced in its native position and the abdominal wall closed with 6–0 suture (35). The outer skin was closed with small wound clips. The animal was kept warm and observed throughout recovery. Wound clips were removed after 10 days.

### Post-transplant Glucose Metabolism

Non-fasting blood glucose levels were checked daily using a glucometer (Contour; Bayer Healthcare LLC, Mishawaka, IN). Body weight was recorded x3/week. Mice with a blood glucose level between 350–450 mg/dl received 0.3–0.5U insulin IP or SC per day. If glucose levels reached 450–600 mg/dl, 0.5–1.0U insulin was given. Additionally, 0.5–1.0 ml sterile saline solution was administered IP prophylactically to hyperglycemic animals daily to avoid dehydration. Islet recipients underwent an intra-peritoneal glucose tolerance test (IPGTT) 2 weeks after transplantation, to determine possible differences in glucose tolerance between groups. Briefly, after a 4 h fast, mice were administered a 2.0 g/kg dose of 10% glucose solution IP, and blood samples were collected at intervals of 0, 15, 30, 60, 90, 120 min after injection (38). Insulin administration was withheld from recipients for 48 h immediately preceding IPGTT tests to insure an accurate test result.

### Histological Examination of Transplanted Islets

Histological examination of the islet graft was performed in mouse recipients of islets alone, islet/HMC or islet/HDF grafts on days 3, 7 and 14 after transplantation. Sections of the native pancreas of the mouse recipients were also immunostained for insulin to confirm absence of β cells therefore corroborating hyperglycemia. Five mice were transplanted for each time-point.

The mice were anesthetized with ketamine/xylazine (100/8 mg/kg) and then euthanized by cervical dislocation. Islet graft-bearing kidneys and the native pancreas were dissected and fixed in 4% paraformaldehyde for 3 h, then transferred to 30% sucrose overnight before embedding in OCT and freezing at −80°C.

Serial sections of 7 μm thickness were cut from each graft and stained for insulin (L6B10, 1:800; Cell Signaling Technology, Danvers, MA), macrophages (Anti-F4/80, 1:100; ABCAM, Cambridge, MA), and cell death (Anti-Caspase 3, 1:800; Sigma-Aldrich). To evaluate vascularization, anti-CD31 antibodies (ABCAM, 1:20) were used on sections obtained from different graft depths at 30μm intervals. To evaluate inflammation, sections were stained with anti-Il-1β (ABCAM, 1:100) and anti-IL-10 (ABCAM, 1:100) antibodies. Antibodies targeting human nuclear antigen (ABCAM, 1:200) were used to identify the human cells (HMCs and HDFs). DAPI (Invitrogen) stained nuclei and, as secondary antibodies, donkey anti-mouse IgG, Alexafluor 488, and goat anti-rabbit IgG, Alexafluor 594 (all from ABCAM, 1:500) were used. Images were captured using a fluorescent microscope using the manufacturer's software (Carl Zeiss Microscopy, Thornwood, NY). Three to 6 transplant regions were analyzed from microscopy images obtained from representative islet recipient mice co-transplanted with either islets alone, HMCs or HDFs. CD31, Il-1β and Il-10 immunostaining were quantified for total fluorescence intensity using MetaMorph imaging software version 7.8.0.0 (MetaMorph, Nashville, TN) and presented as intensity per pixel area. Additionally Caspase 3 and F4/80 positive cells were counted. A relative pixel area was determined by setting the smallest pixel area from these images to a value of 1, with larger images scaling proportionally based on this relative pixel area. Data presented as positive cells per this adjusted pixel area.

### Statistical Methods

Statistical differences between two experimental groups were determined by parametric t-test or Mann-Whitney test, as appropriate. For three groups, a one-way ANOVA (parametric) or Kruskal-Wallis test was performed with a Tukey's multiple comparisons post test. A *p*-value of ≤0.05 with a 95% confidence interval was considered significant.

## Results

### *In vitro* Insulin Secretion and Viability Studies

**Figure 1:**
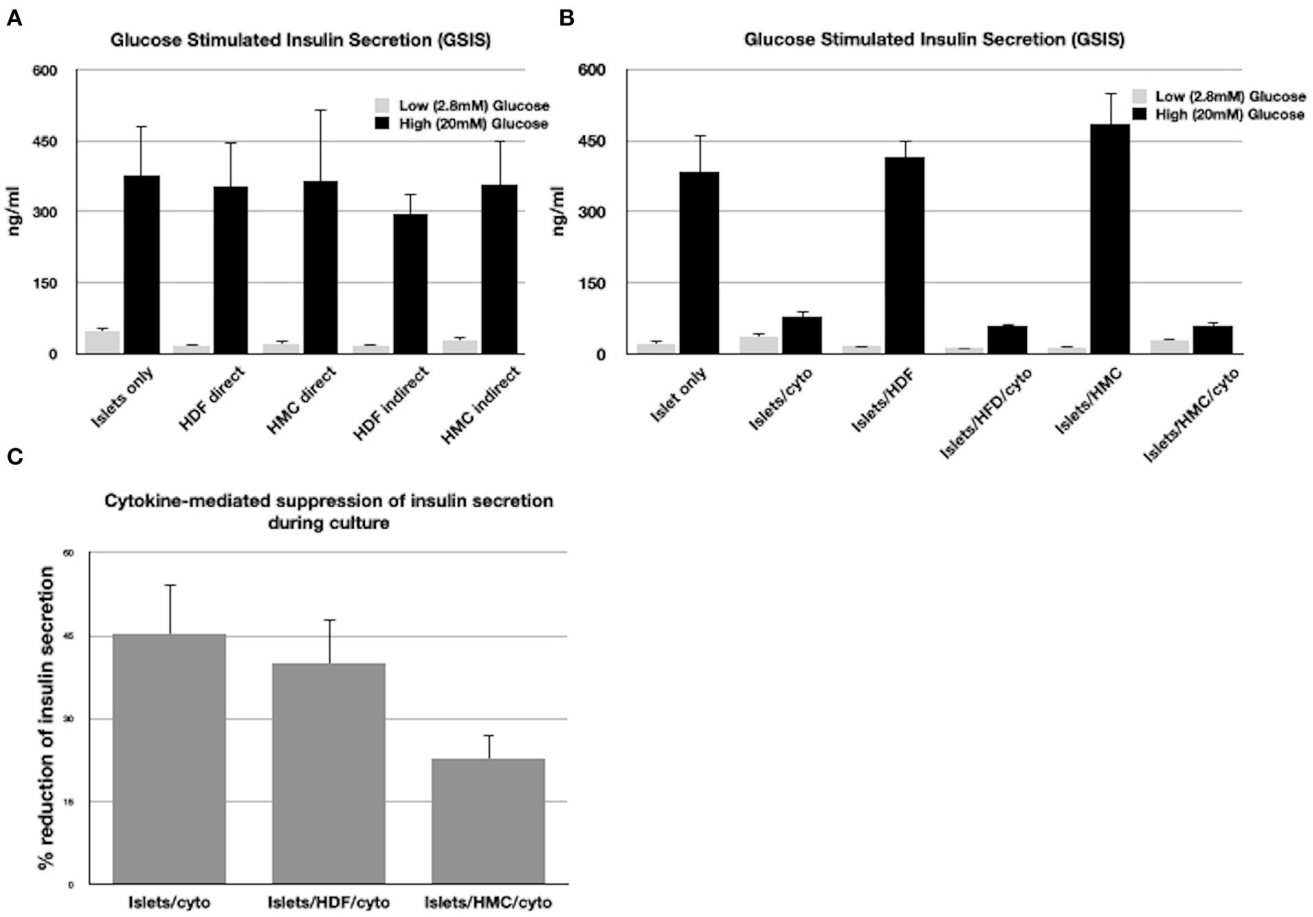
**(A)** GSIS results following exposure of islets only, islets/HMCs or islets/HDF to low and high glucose concentrations. Culture time prior to GSIS testing was 48h. In “direct” cultures, islets and cells were maintained in contact. In “indirect” cultures, islets were separated from cells using Transwell plates. **(B)** GSIS results following 24 h culture of islets alone, islets with HMC, islets with HDF and duplicate groups exposed to inflammatory cytokines (Il-1β, INF-γ, TNF-α). **(C)** Percentage of reduction of insulin secretion during 24 h culture with cytokines of islets alone, islets/HDF and islets/HMC (*p < 0.05 for islets/HMC compared to islets only and islets/HDF). All results consisted of triplicate wells of 25 islets/well per group. Groups with cells were added at a ratio of 500:1 cells/islet.

Figure 1A shows the insulin concentrations released during GSIS by islets co-cultured with HMCs or HDFs through direct (cell-to-cell contact) or through indirect contact (separation *via* Transwell plates) for 48 h. Islets alone were used as control.

All groups exhibited responsiveness to glucose, regardless of the culture settings. No significant difference in insulin secretion was observed, nor of viability, which was >90% in all islets tested under all conditions.

Figure 1B shows the insulin concentrations released during GSIS assay from islets co-cultured with and without HMCs or HDFs after exposure to a cocktail of cytokines for 24 h. Cytokines suppressed stimulated insulin secretion in a similar fashion in all islet combinations. However, as shown in Figure 1C, reduction of insulin secretion during 24 h in culture with cytokines, was significantly less (*p* < 0.05) when islets were co-cultured with HMCs than in islets alone and islets/HDFs, suggesting that HMCs help preserve basal insulin secretion. Following 24 h of cytokine exposure the islet number was unchanged, and viability of the islet cells was >90% with no difference across culture conditions, thus the number of viable islet cells contributing to insulin release was comparable among groups.

### Determination of a Marginal/Minimal Islet Mass Model

The minimal number of islets that allow for normalization of blood glucose levels in diabetic recipients is typically lab/team-dependent. Variables such as islet quality, the approach used to count the islets, donor-recipient species and age, degree of islet purity, site of implantation, culture conditions, and transplantation techniques, all contribute to define a marginal islet mass. In order to define such a mass, mouse islets represent a more consistent product over human or large mammal islets. Mouse islets have more stable qualitative characteristics, whereas human and large mammal islets vary significantly from batch to batch. In order to assess the effect of the HMCs on islet engraftment, it was relevant to first determine a low-enough islet mass to marginally fail in normalizing blood glucose levels in the recipients.

**Figure 2:**
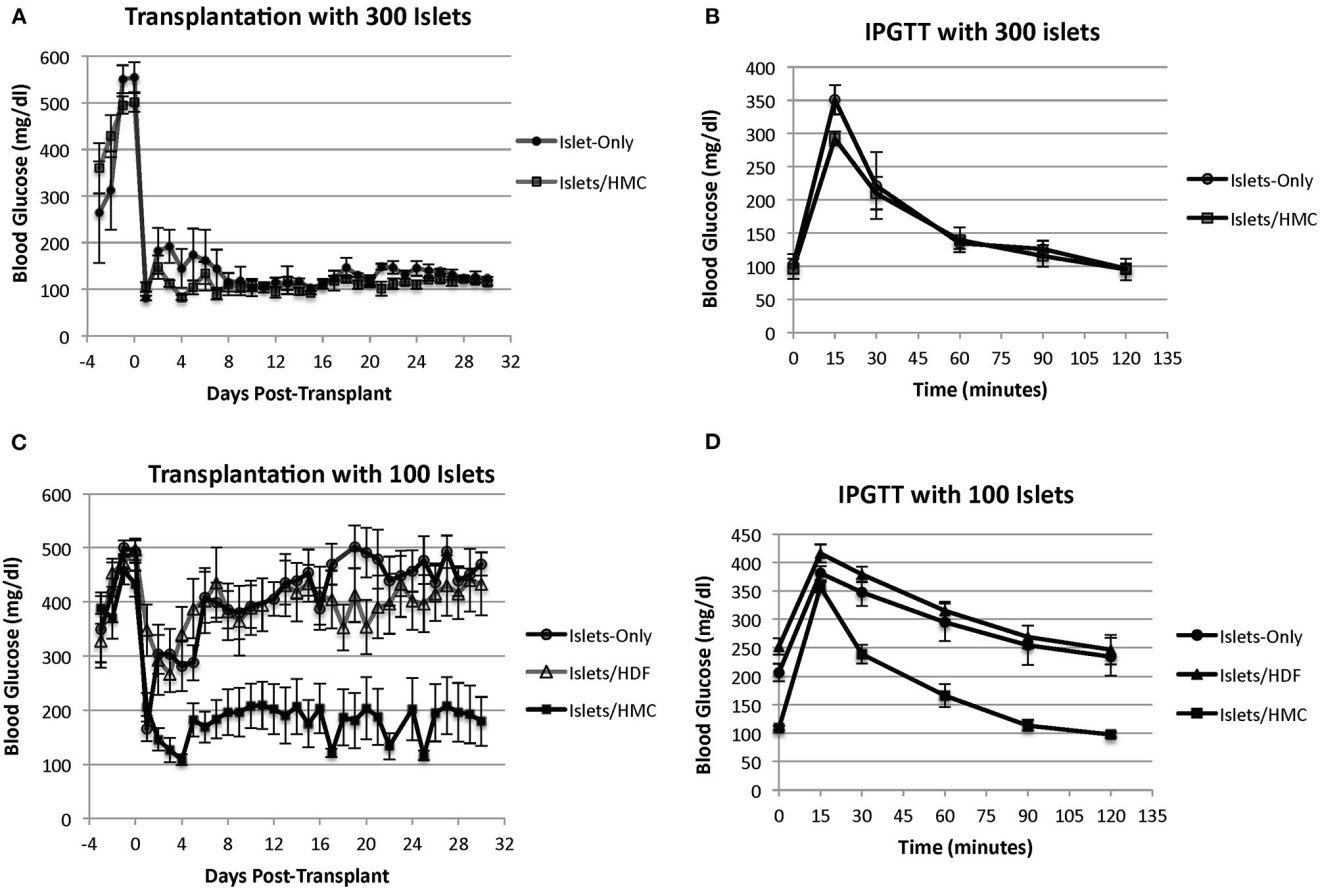
**(A)** Blood glucose levels of mouse recipients following transplantation of 300 islets under the kidney capsule with (*N* = 4) and without (*N* = 3) HMCs. Day 0 = day of transplant. **(B)** IPGTT glycemic curves of the same recipients performed at 2 weeks post-transplant. **(C)** Blood glucose levels of mouse recipients following transplantation of 100 islets. Day 0 = day of transplant. Recipients of islets alone (*N* = 8), islets plus HMCs (*N* = 8), and islets plus HDFs (*N* = 7) Values are means ± SEM. Differences between 100 islets plus HMCs in recipients and the other two control groups are statistically significant (*p* < 0.001). **(D)** IPGTT glycemic curves of the same recipients performed at 2 weeks post-transplant.

We began by transplanting 300 islets per recipient. All animals normalized and the addition of HMCs did not show any effect, for example, on blood glucose normalization (Figure 2A) or on an improved IPGTT curve (Figure 2B). All recipients (HMC: *N* = 4, Islets Only: *N* = 3) normalized their blood glucose after transplantation (<200 mg/dl). Similar results were obtained with 200 islets (HMC:*N* = 3, Islets Only: *N* = 3) (Supplementary Figure 1). Step-wise reduction of the islet mass demonstrated that 100 islets were insufficient to sustain glycemic control. We therefore selected a mass of 100 islets as a marginal mass for the study.

### HMCs-Specificity on Improved Islet Transplantation Outcome

To determine if any human fibroblast-like cell type exerted a specific effect, we compared the outcome of islet transplantation in recipients of islet preparations enriched with HMCs to islets enriched with HDFs. These two cell populations were used in the same numbers, and transplanted in an identical fashion. Viability and morphological integrity of all cell types were indistinguishable prior to transplantation (data not shown).

Figure 2C shows the blood glucose levels in mouse recipients of 100 islets alone (*N* = 8), islets with HMCs (*N* = 8) or islets with HDFs (*N* = 7). Recipients of islets plus HMCs showed a rapid and sustainable drop in blood glucose levels, which stabilized below diabetic levels (~200 mg/dl). No insulin administration was needed. In contrast, the recipients of islets alone or islets/HDFs exhibited a transient decrease in glycemic levels immediately following transplantation, but improved metabolic control was not achieved. This was further emphasized with the results of an improved IPGTT curve for recipients receiving HMCs plus islets over recipients receiving either islets only or islets with HDFs (Figure 2D).

The addition of HDFs to the islets did not provide any beneficial effect on blood glucose concentration. None of the recipients of islets alone or islets with HDFs achieved insulin independence. (Insulin maintenance was withheld from recipients for 48 h before IPGTT).

These results confirm that HMCs confer specific benefits that enhance islet performance. Recipients with normalized glycemic levels showed a consistent and gradual increase in body weight over the 30 day follow-up without exogenous insulin or hydration assistance, further confirming good islet cell function and physiologic glucose metabolism. Recipients without HMCs also maintained body weight but only with daily insulin and hydration therapy (Supplementary Figure 2).

### HMCs Enhance Revascularization and Reduce Inflammation and Apoptosis at the Graft Site

**Figure 3:**
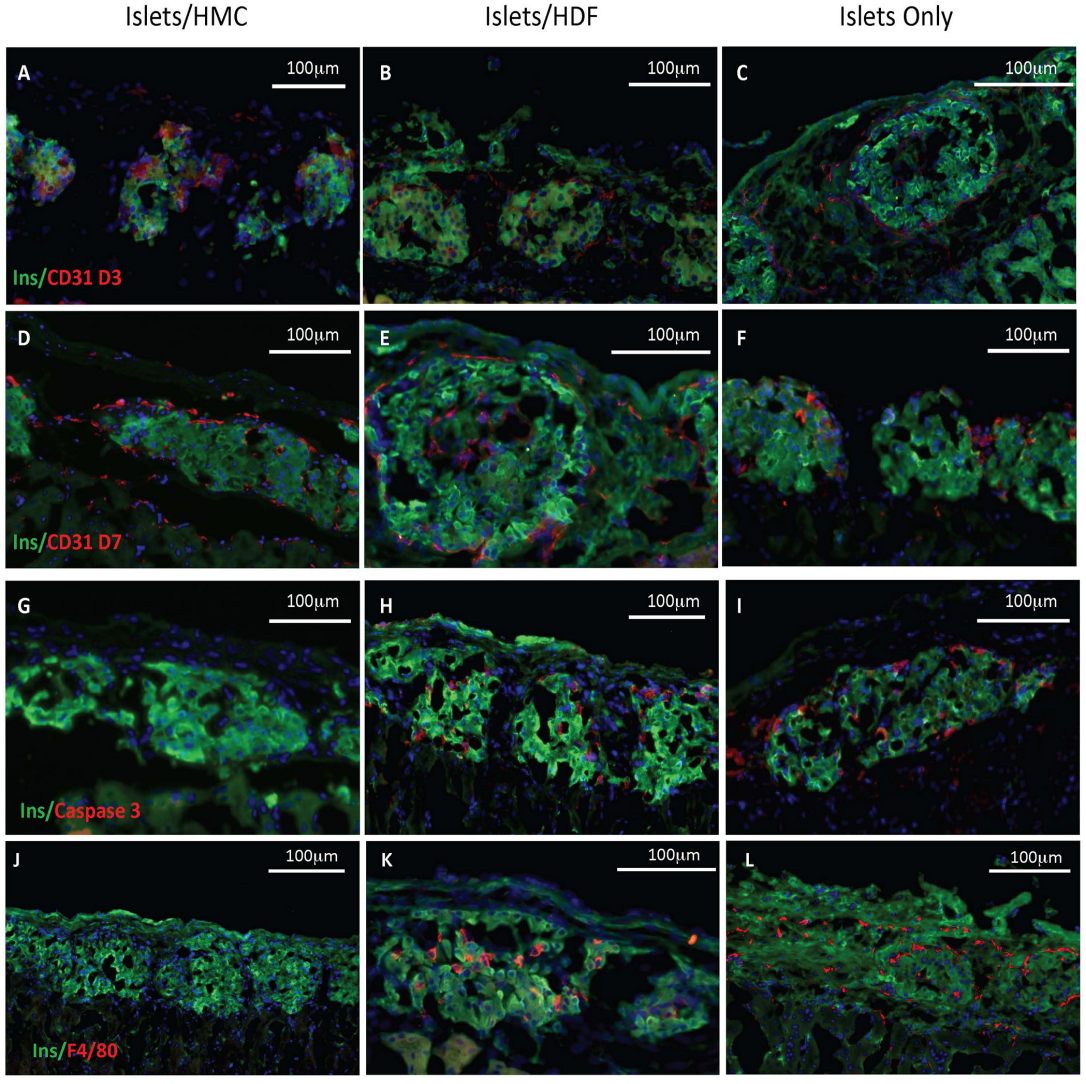
Histological features of the islet grafts. Tissues retrieved from recipients of islets plus HMCs are shown in the left column **(A,D,G,J)**, whereas tissues from recipients of islets plus HDFs are shown in the middle column **(B,E,H,K**) and from Islets Only in the right column **(C,F,I,L)**. Insulin immunofluorescence is in green, nuclei in blue. **(A–F)** CD31, a marker of endothelium is shown in red. **(A–C)** Day 3 post-transplant. **(D–F)** Day 7 post-transplant. **(G–I)** Caspase 3, a marker of apoptosis, is shown in red on day 3 post-transplant. **(J–L)** F4/80, a marker for macrophages, is shown in red on day 7 post-transplant. Magnification 200×.

**Figure 4:**
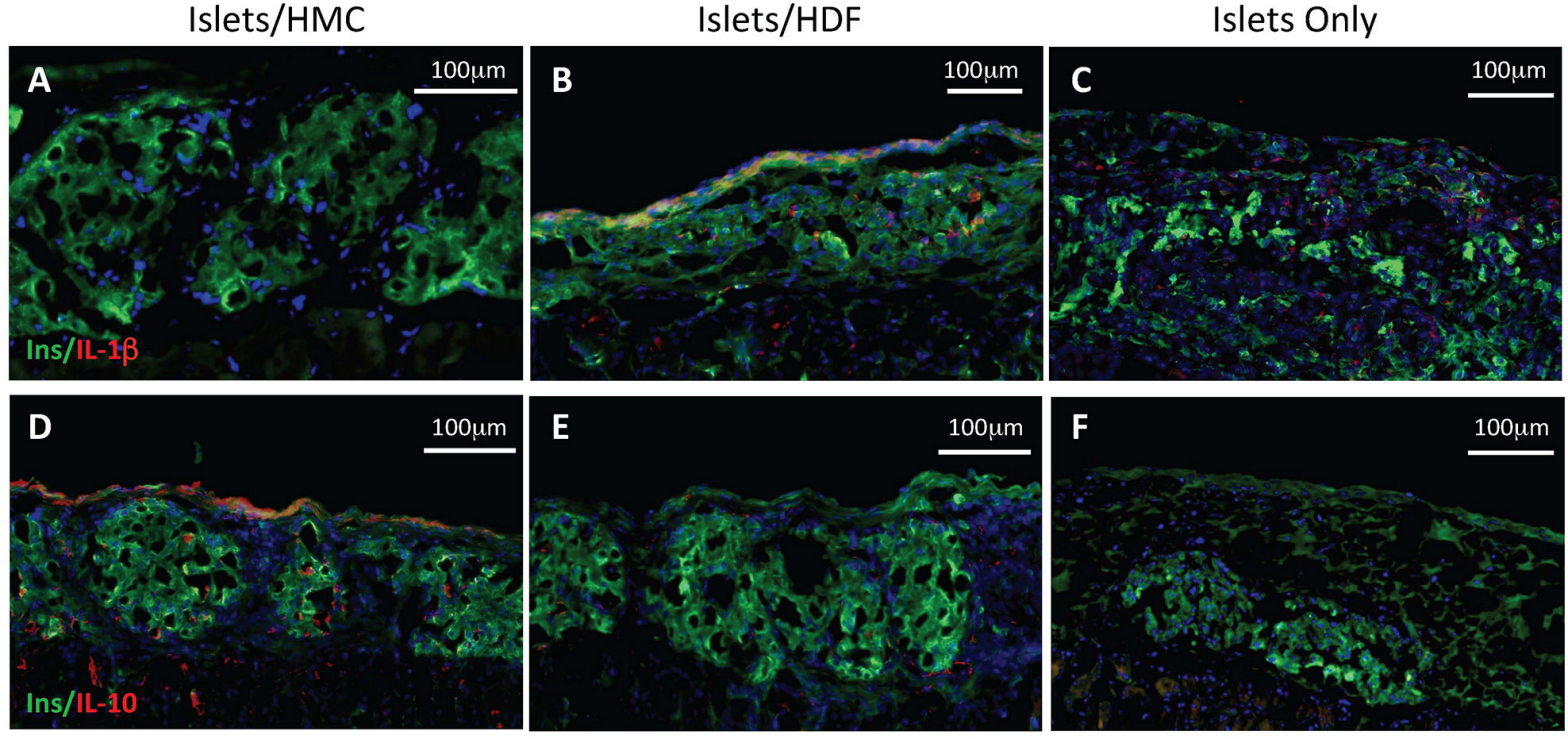
Immunostaining for pro- and anti-inflammatory cytokines. Left panels show representative islets/HMC sections, middle panels show islets/HDF sections, right panels show Islets Only sections. **(A–C)** Staining for pro-inflammatory Il-1β at day 7 post-transplant. **(D–F)** Staining for anti-inflammatory Il-10 at day 3 post-transplant. (Red-cytokine, green-insulin, blue-nuclear DAPI stain) Magnification 200×.

**Figure 5:**
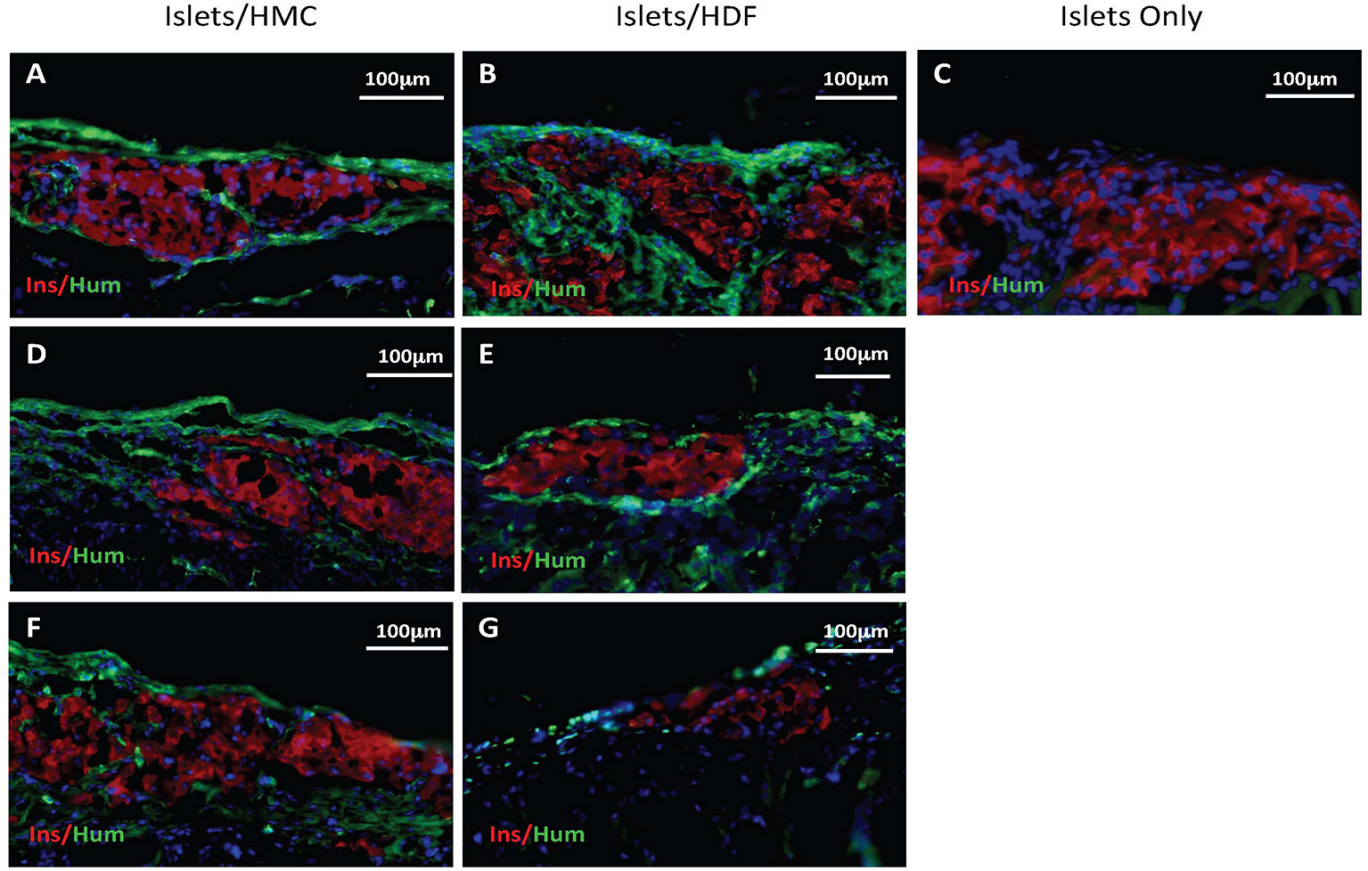
Immunostaining for insulin in red and human nuclear antigen in green as a marker for HMCs or HDFs. Nuclear (DAPI) staining is in blue for all sections. **(A,C)** Sections from day 3 grafts post-transplant. **(D,E)** Sections from day 7 grafts post-transplant. **(F,G)** Sections from day 14 grafts post-transplant. **(A,D,F)** Islets/HMC sections, **(B,E,G)** Islets/HDF sections. **(C)** Section from day 3 post-transplant Islets Only graft. Magnification 200×.

**Figure 6:**
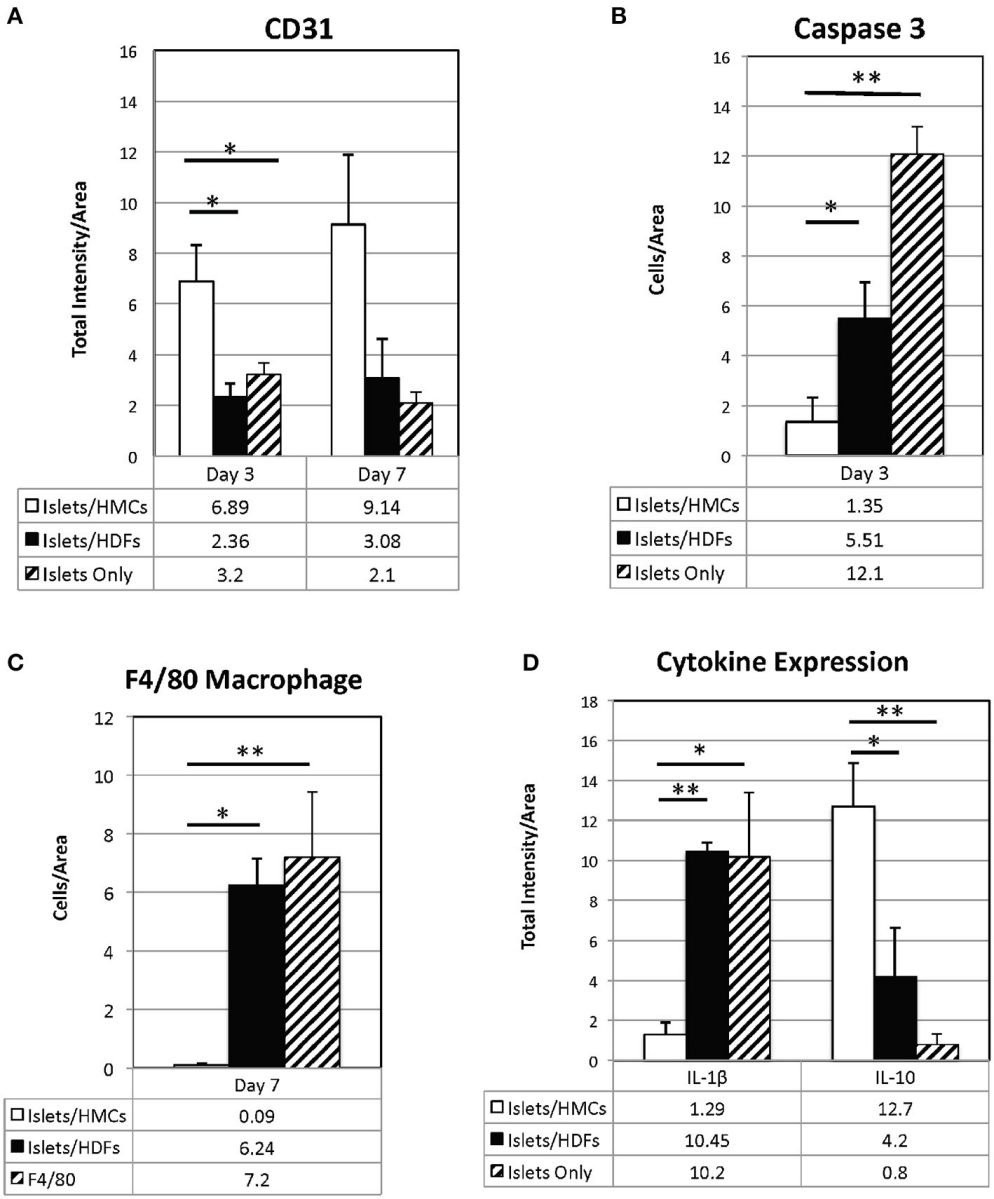
Quantification of immunostaining for CD31 **(A)**, Il-1**β**, and Il-10 **(D)** is expressed as total intensity/pixel area. Caspase 3 **(B)** and F4/80 **(C)** are expressed as cells/ relative pixel area. Graphs show means±SEM. 3 to 6 graft sections from different animals were used in calculating each value. A one-way ANOVA (parametric) or Kruskal-Wallis test was performed with a Tukey's multiple comparisons post-test as appropriate to determine statistical significance. *p < 0.05, **p < 0.01.

To investigate the potential mechanisms of action for the enhanced effects on the glycemic index afforded by HMC co-transplantation, the islet-bearing kidneys co-transplanted with HMCs, HDFs, or Islets Only were collected after euthanasia at day 3, 7, and 14 (*N* = 5 per group per time-point). Sections of the grafts were analyzed by immunofluorescence for markers of vascularization (CD31), apoptosis (Caspase 3), and macrophages (F4/80) (Figure 3). Markers for inflammation (Il-1β and Il-10) are shown in Figure 4. Quantification is shown in Figure 6. A marker of human nuclei was also used to identify the human-derived cells (HMCs or HDFs). Human nuclei were detected in association with insulin-positive cells at all time-points (Figure 5). Insulin staining of the native pancreas confirmed an absence of β cells (Supplementary Figure 3).

As shown visually in Figure 3 and quantitatively in Figure 6A, CD31-positivity was not significantly different by day 7 between islet/HMC, islet/HDF and Islets Only grafts. However, on day 3 islet/HMC grafts showed significantly more CD31 staining than the 2 control groups, suggestive of faster re-vascularization. Day 14 differences were no longer significant.

On day 3, Caspase 3-positive cells were significantly lower in islet/HMC grafts then in islets/HDF or Islets Only grafts (Figure 6B), but not significant at later time-points. Macrophage infiltration was not detected on day 3 in any of the analyzed tissues (data not shown), yet by day 7 macrophages were present in grafts containing islets/HDFs and Islets Only, but not in those containing HMCs (Figure 6C).

Figure 4 represents the evaluation of a pro-inflammatory (Il-1β) and an anti-inflammatory (Il-10) cytokines. At day 7, islet/HMC grafts (Figure 4A) show little evidence of Il-1β whereas the control grafts (Figures 4B,C) show significantly higher positivity (Figure 6D, *p* < 0.01). Concurrently, starting on day 3, islet/HMC grafts (Figure 4D) exhibit a higher Il-10 expression compared to controls at any time-point (Figures 4E,F, 6D).

To verify that human cells were present at the site of the islet grafts, sections were stained (in green) for human nuclear antigen (a marker for HMCs or HDFs) together with mouse insulin (in red) (Figure 5). Human cells were found in all sections of grafts transplanted with either HMCs or HDFs at all 3 time-points. Day 3 sections are shown in A-B, day 7 in D-E and day 14 in F-G (HMC in left column, HDF in middle column). In contrast, staining of Islets Only control grafts (day 3) did not show the presence of human cells (Figure 5C).

Quantitative results obtained from the stained sections are shown in Figure 6. There was significantly greater CD31 vascular staining (Figure 6A, *p* < 0.01) on day 3 post-transplant associated with islets/HMC grafts. Additionally, the islets/HMC grafts showed a noteworthy increase in the anti-inflammatory Il-10 cytokine (Figure 6D-*p* < 0.05). Conversely, there was significantly more apoptosis (Figure 6B: Caspase 3, *p* < 0.05), pro-inflammatory cytokines (Figure 6D: Il-1β, *p* = < 0.001) and activated macrophages (Figure 6C: F4/80 *p* < 0.05) in the grafts *not* protected by HMCs cells.

### Discussion

The enhancing effects of MSCs on islet engraftment have been documented in numerous models (14, 39–41). Based on these studies, the supportive role of MSCs translates into improved glycemic control and a higher rate or faster return to normoglycemia. Such positive *in vivo* effects, however, have been less well-documented using a stringent (and perhaps more clinically relevant) marginal mass model (34, 42–44). To the best of our knowledge, ours is the first marginal islet mass study to demonstrate that the co-transplantation of well-characterized human hESC-based hemangioblast-derived HMCs with islets under the kidney capsule is a critical success factor.

Human MSCs have been typically obtained from adult bone marrow or adipose tissue (45). Adult MSCs, however, present the potential problem of spontaneous dedifferentiation and require pathogen-screening for each donor (46). Moreover, they may require additional steps of preparation prior to achieving the final product suitable for transplantation.

Compared to other sources, more limited data have been accrued for hESC-derived MSCs as supportive cells for islet transplantation. Hajizadeh-Saffar et al., reported that the addition of hESC-MSCs to islets improved islet transplantation outcome, especially if engineered to overexpress vascular endothelial growth factor (VEGF) (34). The advantage of hESC-MSCs over other sources of MSCs lays in their higher proliferative properties, well-defined and stable phenotypical characteristics, and potency. In our study, we used HMCs consisting of a well-characterized cell product, whose phenotype, secretome, and functional properties have been proven stable over multiple passages, cryopreservation, and also across batches. Preclinical biodistribution, safety, and toxicity studies using HMCs indicate that the cells are generally well-tolerated, even without immunosuppression in both murine and canine studies (30–33). These characteristics, together with their high proliferative index, identify them as a highly-suitable clinical cell product.

Ratios from 1–20,000 MSCs/islet have been utilized in previous studies (47, 48), but 500–1,000 MSCs/islet are most commonly used. We chose the lower amount of 500 cells/islet. Whether a greater ratio of HMCs/islet would further enhance the beneficial effects on islet engraftment was not addressed in this study.

The supportive effects of MSCs on islet grafts have been attributed to enhanced and faster revascularization, reduced inflammation and, in allogeneic models, a modulated immune response (13, 14, 39, 49). We have evidence of all three effects in our current study using HMCs. At day 3 after transplant, the grafts with HMCs show a statistically significant increase in CD31 staining suggesting a faster revascularization of the islets than in the non-HMC grafts. By day 7 the difference is not as substantial but it is still increased. Identifying macrophages in the non-HMC grafts suggests a normal immune reaction to foreign tissue that is not seen in the HMC/islet grafts. This ties in with the significantly increased amount of the pro-inflammatory cytokine IL-1β observed in the non-HMC grafts while the grafts with HMC exhibit a statistically significant increase in the anti-inflammatory cytokine IL-10. Moreover, MSCs appear to provide direct stimulation of insulin secretion (43, 45). One mechanism by which MSCs may enhance the glycemic control of transplanted islets is *via* N-cadherin mediated stimulation of insulin secretion (45). While we did not specifically address this mechanism in the current study, it is possible that direct cell to cell contact between the HMCs and beta cells, mediated *via* the N-cadherin pathway, may have contributed to the supportive effects of HMCs that we observe *in vivo*. The mechanisms that support our *in vivo* findings using HMCs confirm and expand on these previous observations.

Histological analysis of the grafts suggested reduced local inflammation and apoptosis, and faster vascularization. Reduced macrophage infiltration, with lower IL-1β and higher IL-10 expression in the graft composed of islets/HMCs, are compatible with the establishment of a favorable microenvironment for islet survival, which was likely better preserved throughout the first critical days.

The enhancing properties of MSCs on insulin secretion, shown by others, may also have contributed to improved graft performance (50). However, our observations yielded some differences from other studies. During a co-culture period of 48 h, HMCs had no primary effect on viability or insulin release. Direct or indirect contact with HMCs also did not affect insulin secretion. Subsequently, when exposed to a cocktail of pro-inflammatory cytokines during a 24 h culture, basal insulin secretion was better preserved in islets/HMC co-cultures, even though blunt insulin release during GSIS (high glucose stimulation) after cytokine exposure was not affected/rescued by HMCs. *In vivo*, the first 24–48 h post-transplantation are critical to the fate of the islet grafts and we therefore speculate that a number of co-factors played roles in protecting the islets by exerting effects within this critical time-period.

Given the histopathological results, and the kinetics of reversal of hyperglycemia in our study, however, it seems more reasonable to attribute the advantage of co-transplantation primarily to an ability to inhibit very early events such as local inflammation, thus reducing islet cell loss, and improving metabolic performance of the graft. This would be in line with recent observations that MSCs improve islet functionality under cytokine stress *in vitro* (51). Our findings also point to a significant improved/faster vascularization of the islets as has been observed by others using different MSCs (40).

Whether the specific experimental conditions, or the intrinsic characteristics of HMCs determine this effect, is not known. De Souza et al. published a meta-analysis of relevant published data regarding islet/MSC culture that reveals inconsistent and often contradictory results between groups (41). This should not be surprising considering the lack of consistency between donors and preparation of MSCs. Most of the published results were based on the use of mouse bone marrow-derived MSCs rather than human-derived cells, and none was based on hemangioblast-derived mesenchymal stem cells (as used in our experiments).

In conclusion, the results of our mouse model demonstrate that co-transplantation of HMCs with islets provides specific support that is beneficial to islet graft survival. Of clinical relevance is that a reduced number of islets was required to consistently restore glycemic balance. Histological examination and data produced *in vitro* help to establish some of the mechanics behind these benefits. Our data correlate with reports that show protective and healing effects that have long been associated with MSCs, if not with all of the mechanisms that have been associated with them.

The HMCs utilized in this study demonstrate characteristics and potency necessary to enhance the ability of islet transplantation, provide a consistent, relatively simple-to-produce, easy-to-use, and highly-scalable MSC product for islet transplantation, and possibly also for other wide-ranging therapeutic uses.

## Data Availability Statement

The raw data supporting the conclusions of this article will be made available by the authors, without undue reservation.

## Ethics Statement

The animal study was reviewed and approved by IACUC Allegheny Health Network.

## Author Contributions

RB, SB, DC, HH, EK, NK, and RL: conceived and planned the experiments. MK and CK: assisted with the technical execution of the experiments. YG and BP: carried out the histological and statistical analysis. MT and NG: contributed to the interpretation of the results and writing of the manuscript. EK, NK, and RL: provided the HMC/HDF for the study. All authors provided feedback on the analysis and preparation of the manuscript.

## Conflict of Interest

EK, NK, and RL are employees of Astellas Institute for Regenerative Medicine, a wholly owned subsidiary of Astellas Pharma engaged in the area of stem cells and regenerative medicine. The remaining authors declare that the research was conducted in the absence of any commercial or financial relationships that could be construed as a potential conflict of interest.

## Abbreviations

GSIS: glucose-stimulated insulin secretion
HDF: human dermal fibroblast
hESC: human embryonic stem cell
HMC: hemangioblast-derived mesenchymal cell
IBMIR: instant blood-mediated inflammatory reaction
IPGTT: intra-peritoneal glucose tolerance test
iPSC: induced pluripotent stem cell
MSC: mesenchymal stem cell.
