# Supplementary figures and images for: Human Hemangioblast-Derived Mesenchymal Stem Cells Promote Islet Engraftment in a Minimal Islet Mass Transplantation Model in Mice
Source: Front Med (Lausanne). 2021 Apr 15;8:660877. doi: 10.3389/fmed.2021.660877 (PMC8081894; doi:10.3389/fmed.2021.660877)

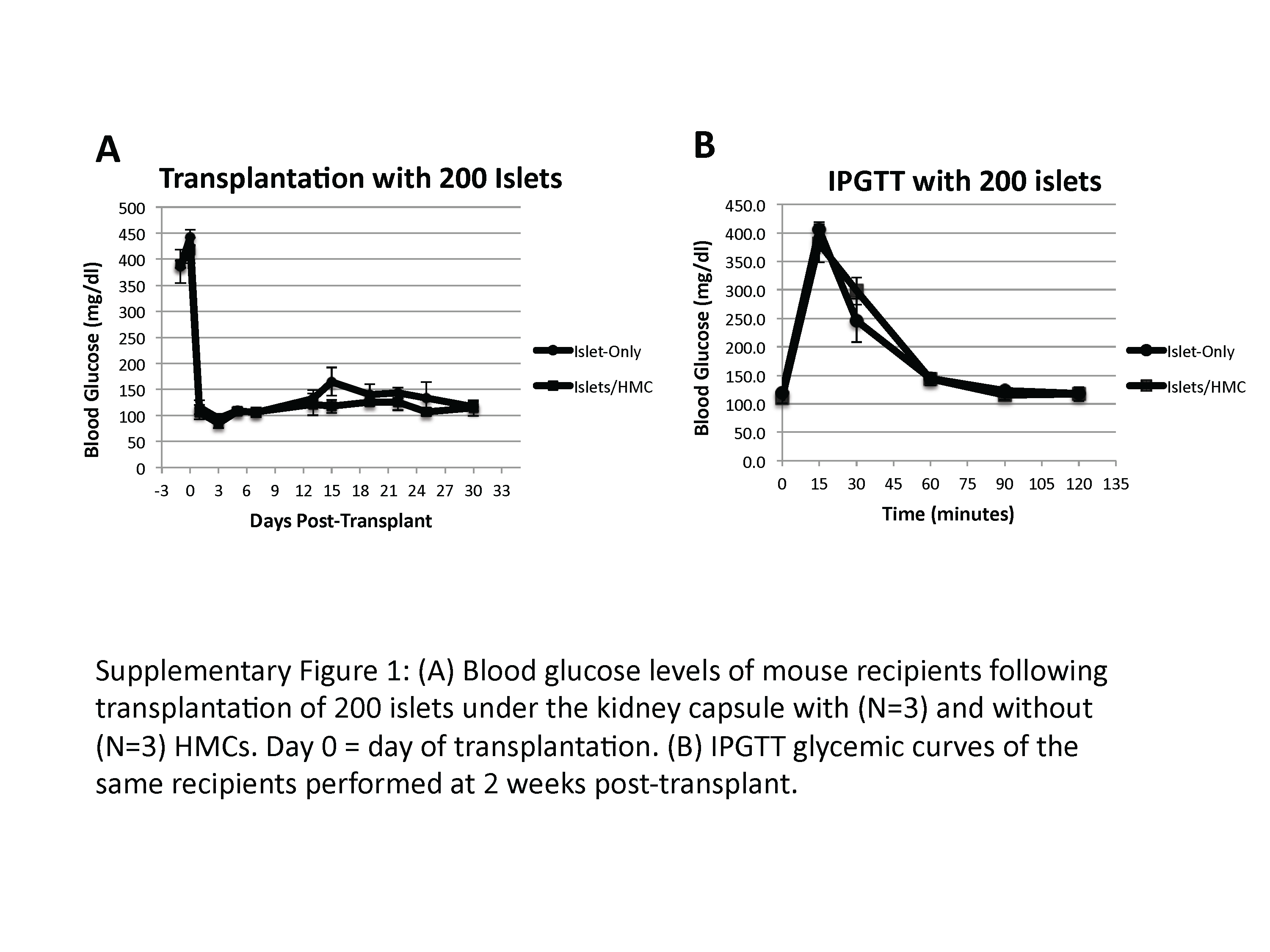

Supplement: Supplementary file 1 [file Image_1.TIFF]

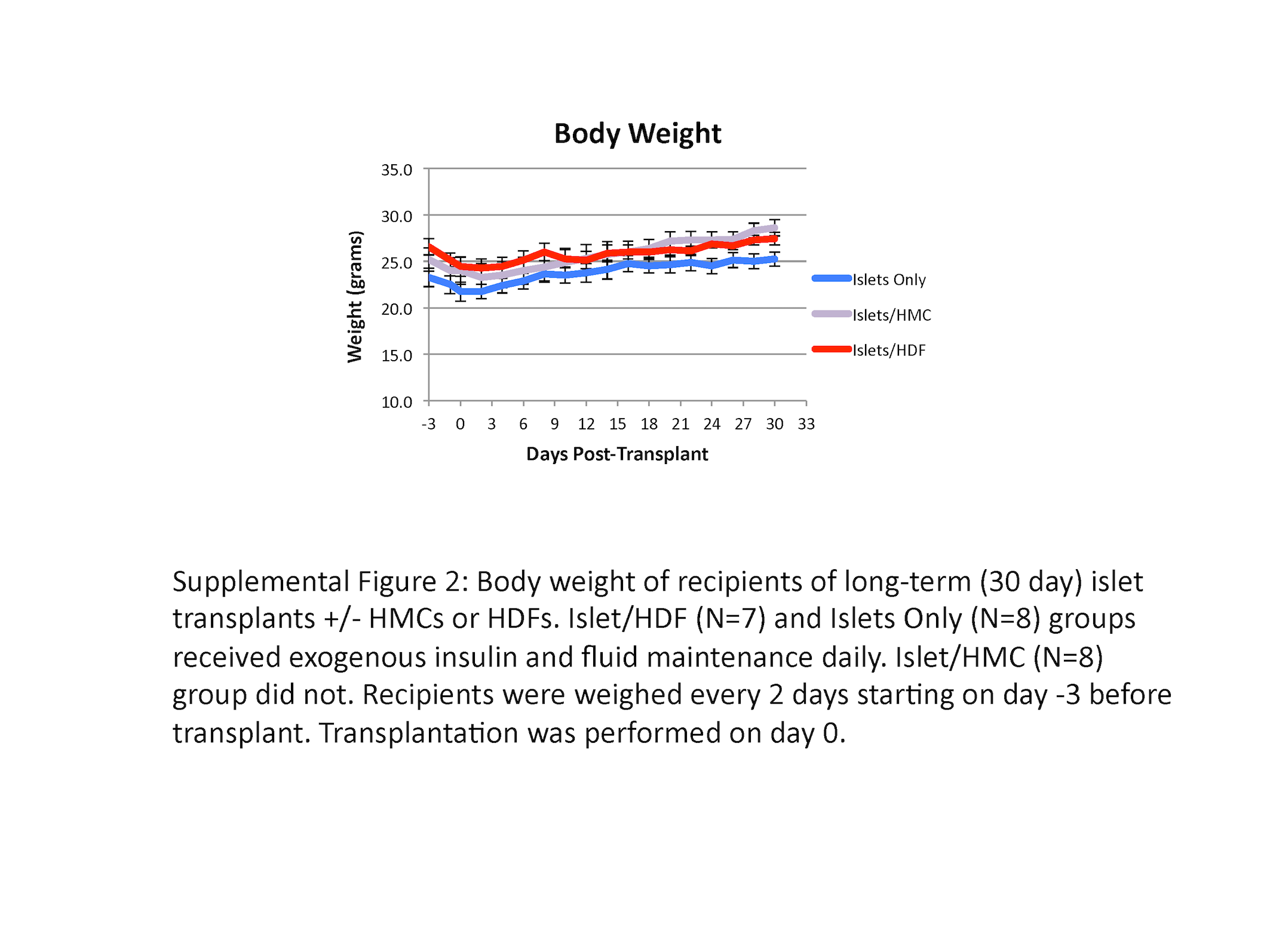

Supplement: Supplementary file 2 [file Image_2.TIFF]

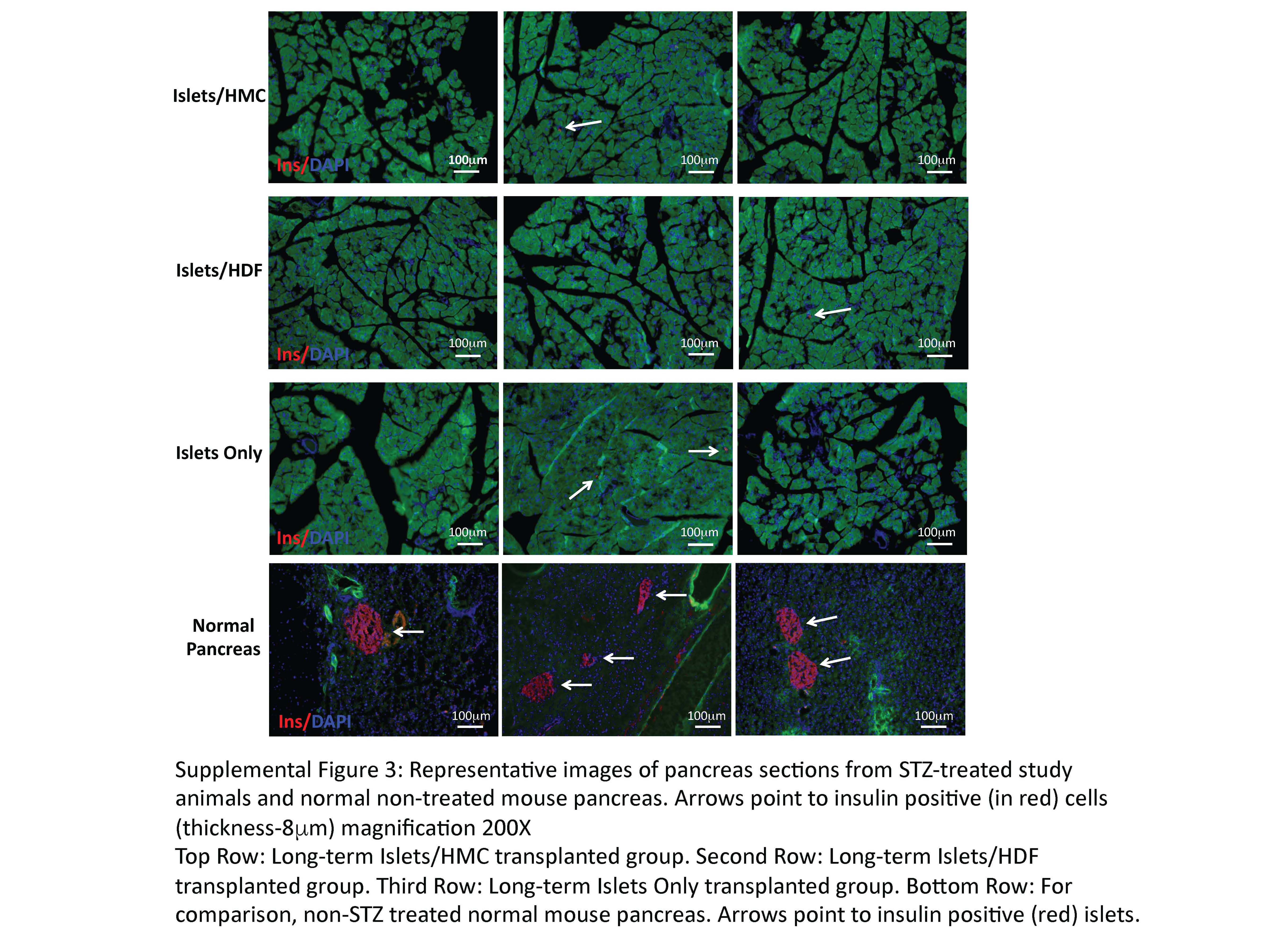

Supplement: Supplementary file 3 [file Image_3.JPEG]
